# The association of reduced bone density with paraspinal muscle atrophy and adipose tissue in geriatric patients: a cross-sectional CT study

**DOI:** 10.3906/sag-1809-48

**Published:** 2019-04-18

**Authors:** Elif Evrim EKİN, Muhittin Emre ALTUNRENDE

**Affiliations:** 1 Department of Radiology, GOP Taksim Training and Research Hospital, Health Sciences University, İstanbul Turkey; 2 Clinic of Neurosurgery, GOP Taksim Training and Research Hospital, Health Sciences University, İstanbul Turkey

**Keywords:** Osteoporosis, bone density, geriatrics, muscular atrophy, adipose tissue

## Abstract

**Background/aim:**

The aim of the study is to examine the relationship among bone density, adipose tissue, and muscle mass with abdominal CT in geriatric patients.

**Materials and methods:**

The study is a retrospective cohort study of patients 65 years and over who underwent abdominal CT for any reason between October 2017 and July 2018. Third lumbar vertebra density, fatty degeneration of the paraspinal muscle, subcutaneous adipose tissue, and mesenteric adipose tissue ratio were evaluated.

**Results:**

A total of 312 patients, 144 females and 168 males, were included in the study. Reduced bone density was found in 237 (76%) patients. Reduced bone density and muscle atrophy was more frequent in females (P < 0.001). Muscle atrophy was found to occur 5.7 times more frequently in cases of reduced bone density (OR, 95% CI = 5.74 (3.27–10.09), P < 0.001). There was no significant relationship found between reduced bone density and subcutaneous adipose tissue thickness or mesenteric adipose tissue ratio (P = 0.073, P = 0.939, respectively).

**Conclusion:**

In the geriatric age group, reduced bone density and muscle atrophy were quite common and were significantly more frequent in women. Furthermore, a strong association between reduced bone density and muscle atrophy was found. No relationship was found between reduced bone density and subcutaneous adipose tissue thickness–mesenteric adipose ratio.

## 1. Introduction

In the geriatric age group, advancing age and decreased activity lead to bone and muscle loss. Age-related bone loss is also dependent on hormonal effects such as changes in sex-steroid hormones, vitamin D, and parathyroid hormone levels (1). Chronic disease and some drugs also play an important role in the pathophysiology of the osteoporosis (2). Along with aging, osteoblast formation decreases whereas lipid storage in the bone marrow increases (1). The pathophysiology of osteoporosis in men has been a popular research topic in recent years. Although male osteoporosis is underrecognized and undertreated, it is considered to be an increasing health problem (3,4). Progressive loss of muscle mass starts around the age of 40 (5). With menopause, muscle mass is reduced by 3% per year, with up to 30%–50% of loss between 40 and 80 years of age (5). Sarcopenia occurs with a loss in muscle mass and muscle strength. Dual-energy X-ray absorptiometry (DXA) and CT are widely used for the diagnosis of osteoporosis and sarcopenia. Paraspinal, psoas, and abdominal wall muscle areas are evaluated in a single transverse cross-section at the L3 level on CT in various studies to diagnosis muscle atrophy (6,7).

The aim of this study was to determine the frequency of reduced bone density in a geriatric patient group with preexisting abdominal CT examinations and to reveal the relationship between adipose tissue and muscle atrophy.

## 2. Materials and methods

This is a retrospective cohort study. Ethics committee approval was obtained for the study. All patients aged 65 years and over who underwent abdominal CT for any reason between October 2017 and July 2018 were sequentially included in the study. 

Patients who were diagnosed with an intraabdominal mass, intraabdominal widespread free fluid, ileus, vertebral tumor, vertebral fracture, or large herniation on the anterior wall of the abdomen were excluded from the study.

A total of 312 patients were included in the study. Retrospectively, our patients were reviewed for chronic diseases using the hospital electronic medical record system. A total of 117 of the 312 patients had significant findings in terms of bone and metabolic diseases: 37 cases of diabetes mellitus (DM), 48 cases of hypertension, 15 cases of DM + hypertension, 9 cases of osteoporosis, 18 cases of asthma, 7 cases of anemia, 11 cases of vitamin D deficiency, 14 cases of hypothyroidism, and 16 cases of depression were found with clinical and laboratory assessments. 

**Figure 1:**
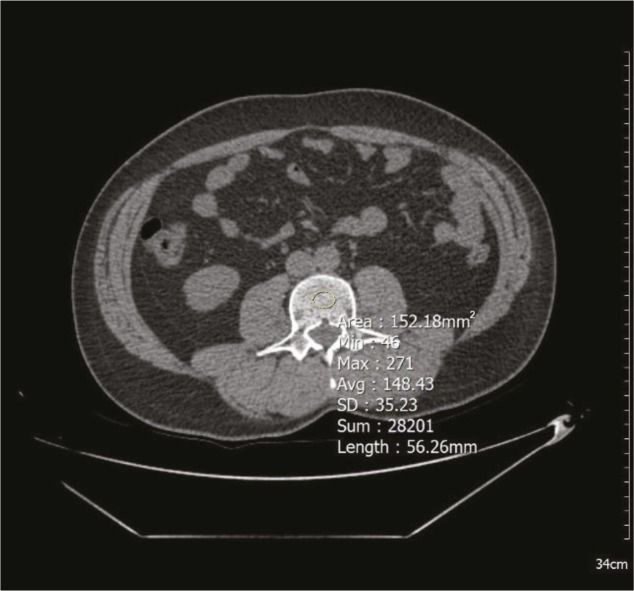
The vertebral density measurement is shown on a transverse CT image. 148 HU, normal vertebral density.

For all shots, 128-slice CT (Optima CT660, GE Healthcare, Chicago, IL, USA) was used. Parameters used for abdominal CT were slice thickness of 3 mm, average 120–130 kV, and 100–130 mAs. All measurements were made on transverse images through the third lumbar vertebra’s (L3) center, using a single oval click-and-drag region of interest (ROI). The vertebral density measurement was performed from the L3 vertebral trabecular region, and the cortex was not included in the measurement area (Figure 1). Average ROI width was 1.5 cm2. Hounsfield unit (HU) values were recorded for each vertebra. The posterior venous plexus, focal vertebral lesions such as hemangiomas, and artifact areas that could affect the value during ROI measurement were avoided. Values of less than 120 HU on the L3 vertebral measurement were accepted as signifying reduced bone density (RBD).

**Figure 2:**
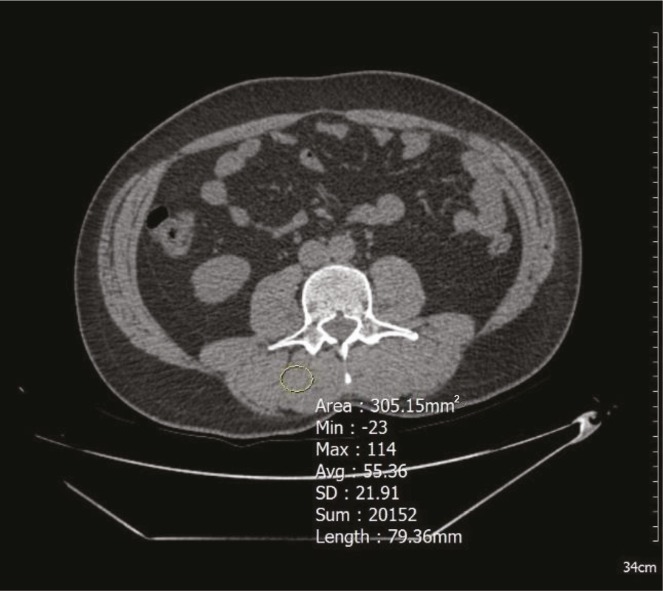
Muscle atrophy is evaluated visually using ROI on the CT image; according to visual evaluation, Grade 0 (normal), and muscle density measurement is 55 HU, which is recorded as normal muscle mass.

Muscle atrophy based on visual appearance and HU value of the muscle were evaluated in the psoas and posterior paravertebral muscles (Figure 2). Following Swash et al. (8), atrophy was graded as follows: Grade 0 (normal); Grade 1 (atrophic), a muscle of reduced area, and/or decreased attenuation; Grade 2, a muscle containing multiple patchy areas of low attenuation; Grade 3, a muscle of generally low attenuation. We considered Grades 1, 2, and 3 to be muscle atrophy and Grade 0 to be normal muscle in our study (8). Garay Mora et al. (9) indicated that less than 30 HU density indicated atrophy in any muscle. In addition to visual assessment (8), muscle density values below 30 HU were recorded as muscle atrophy in our study (9).

**Figure 3:**
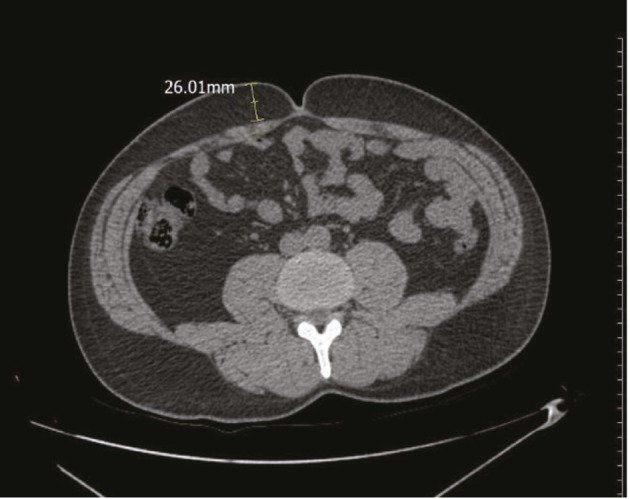
In transverse sections passing through the umbilicus, subcutaneous fat tissue thickness (SCF) was measured from the right side of the umbilicus.

**Figure 4:**
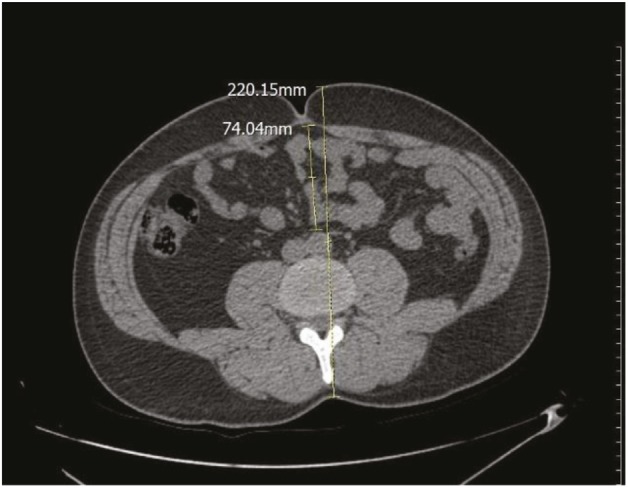
The mesenteric fat tissue thickness ratio was assessed as follows: the ratio of intraabdominal fat tissue thickness (from the anterior wall of aorta to the anterior wall of abdomen) of the entire anterior–posterior abdominal diameter from skin to skin.

In transverse sections passing through the umbilicus, subcutaneous fat tissue thickness (SCF) was measured from the right side of the umbilicus (Figure 3). In the same section, the mesenteric fat tissue thickness ratio (MFR) was assessed as the ratio of intraabdominal fat tissue thickness (from the anterior wall of the aorta to the anterior wall of the abdomen) of the complete anterior–posterior abdominal diameter from skin to skin (Figure 4).

### 2.1. Statistical analysis

Data were tested for normality using the single-sample Kolmogorov–Smirnov test, histogram, Q–Q plots, and box plot graphs. Data are presented as mean, standard deviation, median, minimum, maximum, frequency, and percentage. For the variables with 2 categories (sex and osteoporosis), normally distributed variables were analyzed with an independent-samples t-test, and variables with nonnormal distribution were analyzed with the Mann–Whitney U test. Nominal variables were assessed with the chi-square test. Odds ratio (OR) values and 95% CI were calculated. P < 0.05 was considered significant, and this significance was calculated bidirectionally. All analyses were performed using the NCSS 10 software program (Kaysville, UT, USA).

## 3. Results

A total of 312 patients, 144 females and 168 males, at the age of 65 or above were included in the study. The mean age of all patients was found to be 73.43 ± 7.15 (min–max: 65–95). Mean age was 70 (min–max: 65–95) for females and 72 (min–max: 65–90) for males. Age distribution was found to be homogeneous (P = 0.594, Mann–Whitney U test). RBD was detected in 237 (76%) of 312 patients, while paravertebral muscle atrophy (PVMA) was detected in 197 (63%) patients. The mean subcutaneous fat tissue thickness in all patients was found to be 19 mm (min–max: 3–90).

In terms of RBD, the mean L3 vertebrae density in females was 86 HU (26–169) and it was 104 HU (18–191) in males. RBD was found in 128 of 144 female patients (88.9%), while it was found in only 109 of 168 (64.9%) male patients. RBD was found 4.33 times more frequently in women (OR, 95% CI = 4.33 (2.36–7.9), P < 0.001, Pearson’s chi-square test).

In terms of muscular atrophy, PVMA was found in 112 female patients (77.8%), but in only 85 of 168 male patients (50.6%). PVMA was found to be 3.4 times more frequent in women (OR, 95% CI = 3.4 (2.08–5.62), P < 0.001, Pearson’s chi-square test).

In terms of subcutaneous fat tissue, mean thickness was 25 (3–90) mm in female and 15.5 (3–34) mm in male patients. Subcutaneous fat tissue thickness was higher in females than in males (P < 0.001).

Mean mesenteric fat tissue ratio was 0.33 ± 0.075 in females and 0.34 ± 0.075 in males. There was no sex difference found for MFR (P = 0.196).

Reduced bone density, spinal muscular atrophy, subcutaneous fat thickness, and visceral fat ratio distribution in geriatric patients are given in the Table.

**Table:** Distribution of reduced bone density, spinal muscular atrophy, subcutaneous fat thickness, and visceral fat ratio in geriatric patients (Mann–Whitney U test, Pearson chi-square test).

	Age	Reduced bone density	Spinal muscular atrophy	Subcutaneous fat thickness	Visceral fat ratio
Female (n = 144)	70 (65–95)	88.9% (n = 128)	77.8% (n = 112)	25 (3–90) mm	0.33 ± 0.075
Male (n = 168)	72 (65–90)	64.9% (n = 109)	50.6% (n = 85)	15.5 (3–34) mm	0.34 ± 0.075
P-value	P = 0.594	P < 0.001	P < 0.001	P < 0.001	P = 0.196

In the assessment of RBD–muscle atrophy association, PVMA was detected in 173 patients (73%) with RBD (n = 237). On the other hand, PVMA was detected in 24 patients (32%) with normal bone density (n = 75). Muscle atrophy was 5.7 times more frequent in RBD (OR, 95% CI = 5.74 (3.27–10.09), P < 0.001, Pearson’s chi-square test).

In the assessment of RBD–subcutaneous fat tissue association, SCF was measured as 20 (3–90) mm in patients with RBD and 16 (4–44) mm in patients with normal bone density. No significant difference was found between the subcutaneous fat tissue thickness of RBD patients and that of patients with normal bone density (P = 0.073, Mann–Whitney U test).

In the assessment of RBD–MFR association, MFR was 0.34 ± 0.074 in patients with RBD and 0.34 ± 0.08 in patients with normal bone density. There was no significant relationship between RBD and MFR (P = 0.939, independent-samples t-test).

## 4. Discussion

The highlights of our study are as follows: RBD was detected in 237 (76%) of 312 geriatric patients and was 4.33 times more frequent in female patients (OR, 95% CI = 4.33 (2.36–7.9), P < 0.001). On the other hand, RBD affected more than half of the males in the geriatric population. In 312 geriatric patients, PVMA was detected in 197 patients (63%) and was 3.4 times more frequent in females (OR, 95% CI = 3.4 (2.08–5.62), P < 0.001). Muscle atrophy was 5.7 times more frequent in RBD patients (OR, 95% CI = 5.74 (3.27–10.09), P < 0.001). There was a strong association between RBD and muscle atrophy in our study. Sarcopenia and osteoporosis have been closely linked in the recent literature, especially affecting the geriatric age group. Osteosarcopenia is a new terminology used for the diagnosis of both osteoporosis and sarcopenia in older patients (10). In our study, RBD and muscle atrophy detected on CT may not fully meet the definition of osteosarcopenia. On the other hand, the risk of osteosarcopenia can be determined with a simple evaluation of the preexisting CT and further examination may be recommended. Murray et al. (11) indicated that it is possible to diagnose osteoporosis, obesity, and sarcopenia on abdominal CT, and that diagnosing these is beneficial to the patient. 

DXA is routinely used for the diagnosis of osteoporosis affected by degenerative changes. This study shows a significant limitation of DXA (12,13). Surgical clips, barium sulfate, other metal objects, osteophytes, syndesmophytes, compression fractures, and aortic calcification falsely elevate BMD (14). Marinova et al. (15) reported that vertebral density measurement on CT was sensitive in terms of detecting osteoporosis and superior to DXA. Li et al. (16) recommended that radiologists examine and report osteoporosis on routine abdominal CT. In addition, Zaidi et al. (17)noted that HU value measurement is a useful and practical technique for assessing bone quality that could be reported by a radiologist in all patients with preexisting abdominal CT scans. Pickhardt et al. (18), in their comprehensive series using CT and DXA, determined that their threshold values for osteoporosis at vertebrae L1 to T12 ranged from 110 to 120 HU, whereas normal vertebral density was determined as 200 HU or higher. Values over 200 HU are reported to be accepted as normal and not requiring DXA (18). According to those studies, we evaluated values below 120 HU at the L3 vertebra level as osteopenia/osteoporosis (reduced bone density = RBD). In our study, the diagnosis of muscle atrophy on CT was based on the measurement of density (8) and visual assessment (9) of the psoas and paraspinal muscles at L3 level. 

There have been various studies examining the association between osteoporosis and adipose tissue in the literature. It has been reported that there is a mechanism increasing adipogenesis in stem cell physiology and reducing osteoblastogenesis and bone formation with increasing age (1). Another common hypothesis is that adipose tissue and obesity function as physical barriers and induce osteoblastogenesis and increase bone formation (19–21). It has been reported that android type obesity with visceral predominance has more risks than gynoid type obesity with subcutaneous predominance (22). Measurement of subcutaneous fat tissue and mesenteric fat tissue at the L3 level using additional software during the CT scan can be found in the literature (23–25). We measured the paraumbilical subcutaneous fat tissue thickness and MFR at the L3 level; subcutaneous fat tissue thickness was found to be higher in females than in males (P < 0.001). Subcutaneous fat tissue thickness was not significantly different between patients with and without reduced bone density (P = 0.073, Mann–Whitney test). There was no significant relationship between bone density and MFR (P = 0.939). In our study, there was no significant relationship found between reduced bone density, subcutaneous fat tissue, and mesenteric fat tissue, suggesting that visceral lipidosis may not be a risk factor for reduced bone density. 

As a limitation of this study, in terms of nonvolumetric examination, we may have failed to determine the actual increase in visceral lipidosis with the practical measurement method. With mesenteric fat tissue measurement, we tried to implement an easy-to-use method that can be applied during preexisting CT evaluation and does not require additional cost or time. It is recommended that this method be compared with volumetric measurement of fat tissue in CT with a new study. Another limitation is that body mass index was unknown for the patients in this retrospective study.

In conclusion, our results demonstrated that reduced bone density and muscle atrophy were frequently seen in the geriatric population, significantly more frequently in women. Furthermore, a strong association was found between reduced bone density and muscle atrophy. No relationship was found between reduced bone density and subcutaneous fat tissue thickness–mesenteric adipose ratio.

## Acknowledgment

Thanks to Sevim Purisa for statistical evaluation.
